# CRISPR/Cas9-mediated *Serine protease 2* disruption induces male sterility in *Spodoptera litura*

**DOI:** 10.3389/fphys.2022.931824

**Published:** 2022-08-03

**Authors:** Honglun Bi, Xia Xu, Xiaowei Li, Yaohui Wang, Shutang Zhou, Yongping Huang

**Affiliations:** ^1^ State Key Laboratory of Cotton Biology, School of Life Sciences, College of Agriculture, Henan University, Kaifeng, China; ^2^ Institute of Sericulture and Tea Research, Zhejiang Academy of Agricultural Sciences, Hangzhou, China; ^3^ Key Laboratory of Insect Developmental and Evolutionary Biology, CAS Center for Excellence in Molecular Plant Sciences/Institute of Plant Physiology and Ecology, Shanghai, China

**Keywords:** *Spodoptera litura*, *Ser2*, male sterility, pest control, CRISPR/Cas9

## Abstract

Male fertility is essential for reproduction and population growth in animals. Many factors affect male fertility, such as courtship behavior, sperm quantity, and sperm motility, among others. Seminal Fluid Proteins (SFPs) are vital components of seminal fluid in the male ejaculate, which affect male fertility, sperm activation, and female ovulation. However, the knowledge of SFPs is insufficient; the function of many SFPs remains unknown, and most described functions were mainly characterized in *Drosophila* or other laboratory models. Here, we focus on the *Serine protease 2* (*Ser2*) gene in the lepidopteran pest *Spodoptera litura*. The *Ser2* gene was specifically expressed in male adults. Disruption of the *Ser2* gene mediated by CRISPR/Cas9 induced male sterility but females remained fertile. PCR-based detection of the next-generation mutants showed that male sterility was stably inherited. The qRT-PCR analysis of *SlSer2* mutants showed that motor protein family genes and structural protein family genes were down-regulated, while protein modification family genes were up-regulated, suggesting that *SlSer2* may be involved in sperm movement and activity. These results demonstrate that *Ser2* is an important component of SFPs in seminal fluid and was identified for a useful sterile gene for pest control that may lead to new control strategies for lepidopteran insect pests such as *S. litura*.

## Introduction

The common cutworm, *Spodoptera litura* (Lepidoptera: Noctuidae) is one of the most destructive phytophagous pests of crops such as tea, tobacco, and vegetables, which causes serious losses of yield and quality of crops in China and other Southeast Asian countries (Rao et al., 1993; Qin et al., 2004; Meagher et al., 2008; Muthusamy et al., 2014). Chemical insecticides are the most commonly used control method for *S. litura* (Ayyanna et al., 1982). Resistance to chemical insecticides has become a serious problem in insect pests including *S. litura* (Ahmad et al., 2008; Ahmad et al., 2009; Shad et al., 2010). Moreover, incorrect use of certain pesticides has very serious implications for food security and human health, raising awareness of their eco-environmental and human impacts (Kaur et al., 2014; Rehan et al., 2014). Therefore, alternative species-specific and co-friendly pest management strategies are needed.

The CRISPR/Cas9 system is an effective genome editing tool with potential application in pest management (Wang et al., 2013; Alphey, 2014; Esvelt et al., 2014; Gantz et al., 2015; Alphey, 2016). CRISPR/Cas9 has been used to edit the genomes of numerous eukaryotic organisms (Cong et al., 2013; Hwang et al., 2013), model insect species in the orders Diptera (Bassett et al., 2013; Hall et al., 2015) and Coleoptera (Gillies et al., 2015). In Lepidoptera, CRISPR/Cas9 has been used to analyze gene functions (Liu et al., 2017; Xu et al., 2017; Zeng et al., 2017), to enhance antiviral responses (Chen et al., 2017), to use the silkworm as a bioreactor for important protein products (Xu et al., 2018), and to control female-specific embryonic lethality (Zhang et al., 2018). In *S. litura*, the CRISPR/Cas9 system has worked efficiently to investigate some gene functions (Bi et al., 2016; Zhu et al., 2016; Bi et al., 2019; Du et al., 2019). But how best to use this powerful biotechnology and select suitable and efficient targeted genes to control *S. litura* is a significant problem.

The male seminal fluid is a complex medium, that contains many molecules and complex components, such as seminal fluid proteins (SFPs), produced mainly by sex accessory glands (Poiani et al., 2006; Sirot et al., 2014; McGraw et al., 2015). In the mating behavior of insects, SFPs are transferred from males to females through ejaculation, which has possible benefits including sperm capacitation, sperm competition and fertilization, and plays a crucial role in reproductive success (Chapman et al., 2001; Avila et al., 2011; Denis et al., 2017; Taniguchi et al., 2018; Karr et al., 2019). In *Drosophila melanogaster*, RNAi-mediated knockdown of a type of SFP, Seminase, a predicted serine protease, results in a decrease of eggs and an inability to store sex peptides (LaFlamme et al., 2012). Seminase initiates the protease cascaded signaling pathway by causing proteases to hydrolyze accessory gland proteins (Acps), thus participating in the early regulatory process of the post-mating processes (Laflamme and Wolfner, 2013). Previous studies have shown that serine protease is an important enzyme that promotes sperms to produce energy resources for sperm motility, and its absence affects fertilization success (Nagaoka et al., 2012). Using CRISPR/Cas9 technology to knockout of one of the serine protease gene, *Serine protease 2* (*Ser2*), led to male sterility but did not affect female sterility in *Bombyx mori* and *Plutella xylostella* (Xu et al., 2020).

Here, we investigate the function of the *Ser2* gene in *S. litura*. Using the CRISPR/Cas9 genome editing system, we successfully knocked out the *Ser2* gene. *Ser2* disruption induced male specific sterility in adults with few normal hatched individuals in the next generation. The novel phenotype of male specific sterility showed *Ser2* was an important gene in the sperm development process in *S. litura*. The Sterile insect technology (SIT), which needs to release the sterile insects into the wild and mate with wild type insects that induces the insects sterility, is one of valuable and environmentally friendly pest control approach in lepidopteran and dipteran insects (Tan et al., 2013). Our data indicates that *SlSer2*, which regulates the fertility of male adults and could decrease the population quantity of pests, through releasing male or female mutants, is a potential male specific sterility gene for using in the control of *S. litura* and other lepidopteran pests.

## Materials and methods

### Insect strains and rearing

A laboratory strain of common cutworm, *S. litura*, was obtained from the College of Plant Protection, Nanjing Agricultural University*.* Larvae were provided with an artificial diet (Supplementary Table S1) and were kept at 26°C with 80% relative humidity and a 12:12 light:dark photoperiod. Adults were fed 10% honey and kept at 25°C with 80% relative humidity (Bi et al., 2019).

### Cloning of *SlSer2* and conservative analysis

To identify the *SlSer2* gene sequence, based on a homologous gene sequence aligning approach, the *B. mori Ser2* sequence (NP_001153675.1, NCBI) was used to search for the *S. litura* homolog *Ser2* sequence using local Protein Basic Local Alignment Search Tool (BLAST) of *S. litura* protein database. According to the genome sequence of *S. litura* (Cheng et al., 2017), the related sequence of *SlSer2* was found and designed primers to amplify, used the Polymerase Chain Reaction (PCR).

Total RNA was isolated from fifth instar larvae using Trizol Reagent (Invitrogen, Carlsbad, CA, United States) and treated with RNase-free DNase I (Ambion, Austin, TX, United States) according to the manufacturer’s protocol. cDNAs were synthesized with the Omniscript reverse transcriptase kit (Qiagen, Hilden, Germany) in a 20-μl reaction mixture containing 1 μg total RNA per the manufacturer’s instruction. *SlSer2* cDNA fragments were amplified by PCR with the following pair of primers (Supplementary Table S2). PCR was carried out using KOD plus polymerase (TOYOBO, Osaka, Japan) under the following conditions: 98°C for 2 min, followed by 30 cycles at 98°C for 30 s, 55°C for 30 s, and 68°C for 1 min, and an elongation phase at 68°C for 10 min. Amplified products were sequenced after cloning into a PJET1.2-T vector (Fermentas, Burlington, ON, Canada).

The multiple alignment and conservative analysis were used DNAMAN 8.0 software, including the putative SER2 protein of *S. litura* and the other eight lepidopteran SER2 or homologous amino acids sequences (Supplementary Table S2). The GenBank accession numbers of the protein sequence are as follows: *B. mori* (NP_001153675.1), *Agrius convolvuli* (BAK52270.1), *Samia ricini* (BAL04890.1), *Pieris rapae* (XP_022113521.1), *Helicoverpa armigera* (XP_021195380.1), *Papilio machaon* (XP_014359308.1), and *P. xylostella* (XP_011553524.1), *Hyphantria cunea* (Li L. et al., 2022).

### Expression profile analysis of *SlSer2*

To investigate the spatio-temporal distribution of *SlSer2*, total RNA was isolated from each developmental stage, including eggs, the first day of each larval instar, pupae (P), adults (A), and tissues/organs in the male on the third day of the fifth instar larval (L5D3) stage, including head, the epidermis (EPI), fat body (FB), trachea, foregut (FG), midgut (MG), hindgut (HG), and testis (TE), using Trizol reagent (Invitrogen, Carlsbad, CA, United States) and treated with RNase-free DNase I (Ambion, Austin, TX, United States) according to the manufacturer’s protocols. cDNAs were synthesized using the Omniscript reverse transcriptase kit (Qiagen, Hilden, Germany) in a 20-μl reaction mixture containing 1 μg total RNA from a mixture of equal amounts of three RNA samples from each developmental stage. qRT-PCR analysis for *Ser2* was performed using a SYBR Green Realtime PCR Master Mix (Thermo Fisher, Waltham, MA, United States) on an Eppendorf Real-time PCR System. The PCR conditions were as follows: initial incubation at 95°C for 5 min, 35 cycles at 95°C for 15 s and 60°C for 1 min qPCR reactions were carried out using gene-specific primers to amplify a 208-bp fragment. Another pair of primers, Actin-qF and Actin-qR (Supplementary Table S2), was used to amplify a 159-bp fragment of *SlActin* as an internal control.

### *In vitro* transcription of Cas9 mRNA and sgRNA

Two 23-bp sgRNAs were selected to target *SlSer2*. Each sgRNA was sub-cloned into the 500-bp linearized CloneJet PJET1.2-T vector (Thermo Fisher, Waltham, MA, United States) upstream of the protospacer adjacent motif (PAM) sequence to allow sgRNA expression under the control of the T7 promoter. The sgRNA was synthesized *in vitro* with a MEGAScript T7 kit (Ambion, Austin, TX, United States) according to the manufacturer’s instructions. Cas9 mRNA was synthesized *in vitro* using an mMESSAGE T7 Kit (Ambion, Austin, TX, United States) with a PTD1-T7-Cas9 vector as the template (Wang et al., 2013) according to the manufacturer’s instructions.

### Microinjection of embryos

Female *S. litura* moths were allowed to lay eggs on transparent plastic bags. A previously reported microinjection method was employed (Bi et al., 2016). Within 1 h after oviposition, eggs were injected on the lateral side with 10 nl of a mixture containing 300 ng/μl of *Cas9* mRNA and 150 ng/μl of each sgRNA. After injection, eggs were incubated in a humidified chamber at 25°C for 4 days until hatching.

### Genomic DNA extraction and identification of mutagenesis

The genomic DNA was extracted from newly hatched larvae and adult legs, incubated with proteinase K, and purified via a standard phenol: chloroform extraction and isopropanol precipitation, followed by RNaseA treatment. PCR was carried out to identify *SlSer2* mutant alleles using primers F2 and R1 (Supplementary Table S2) spanning the target site in *SlSer2*. The PCR conditions were as follows: 98°C for 2 min, followed by 35 cycles of 94°C for 10 s, 55°C for 30 s, and 72°C for 1 min, followed by a final extension period of 72°C for 10 min. The PCR products were cloned into pJET1.2-T vectors (Fermentas, Burlington, ON, Canada) and sequenced. The adults and eggs of *SlSer2* mutants were photographed with a digital stereoscope (Nikon AZ100, Tokyo, Japan).

### Mating behavior analysis and hatchability assay

In order to evaluate mating behavior and hatchability of mutants, the mutants of *SlSer2* male and female were crossed with mutant moth and virgin wild type male or female moths. Five pairs of moths were collected for one group. Mating behavior analysis and hatchability assay of each group were repeated three times. The behavioral assays were performed in the transparent plastic bag for one pair. After female moths laid eggs, the eggs of each pair were collected and incubated in a humidified chamber at 25°C for 4 days until hatching. The morphological investigations of mating behavior and egg masses were used the microscope (Nikon AZ100, Tokyo, Japan).

### Statistical analysis

A two-tailed Student’s *t*-test was used to analyze differences between wild-type and mutant individuals. Three independent replicates were used for each treatment and error bars showed the means ± SEM.

## Results

### Identification and characterization of the *SlSer2* gene

**FIGURE 1:**
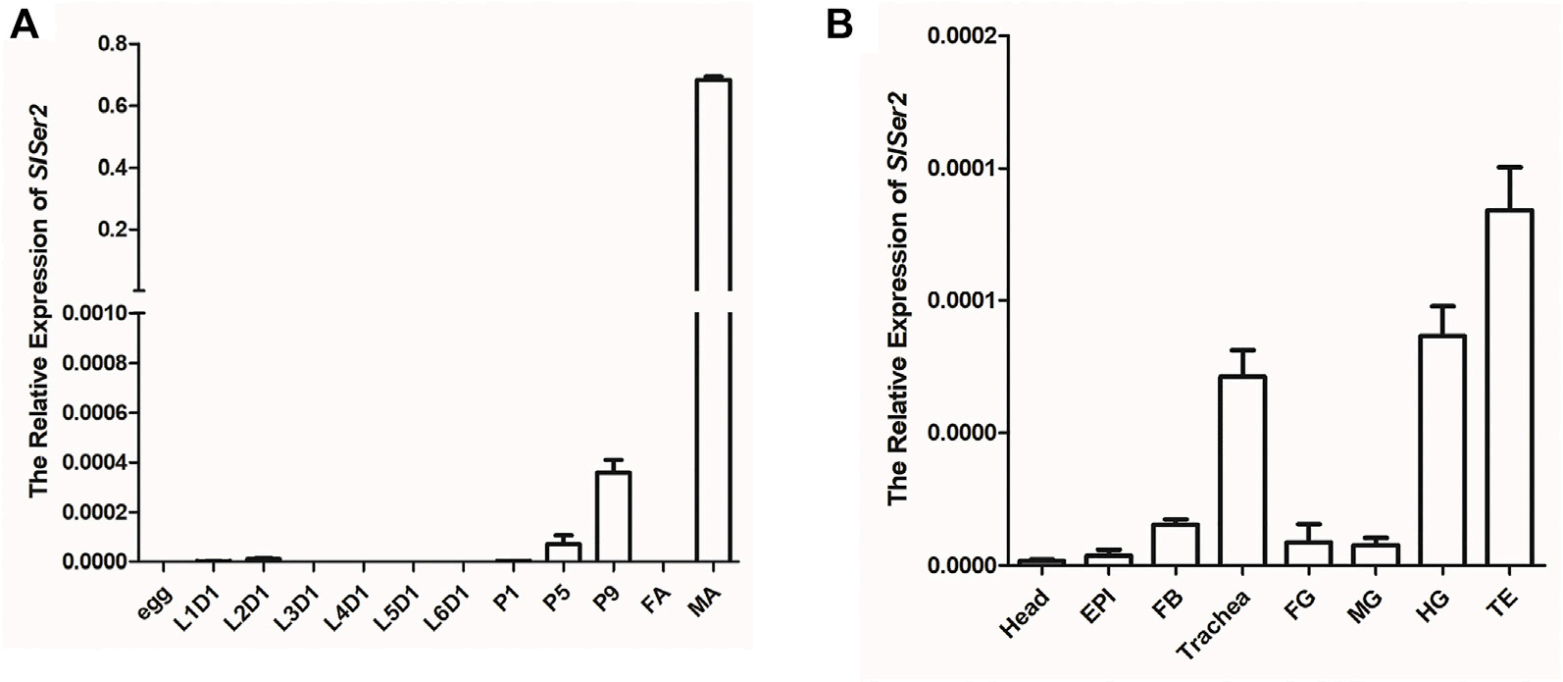
The expression patterns of *SlSer2* in different developmental stages and various tissues. **(A)** Relative mRNA expression of *SlSer2* in different developmental stages, including egg, the first day of each instar of larva (L1D1, L2D1, L3D1, L4D1, L5D1, and L6D1), pupa (P1, P5, and P9), female adult (FA), and male adult (MA). **(B)** Relative mRNA expression of *SlSer2* in the eight reproductive tissues at the third day of the fifth instar larval (L5D3) stage, including head, epidermis (EPI), fat body (FB), trachea, foregut (FG), midgut (MG), hindgut (HG), and testis (TE).

**FIGURE 2:**
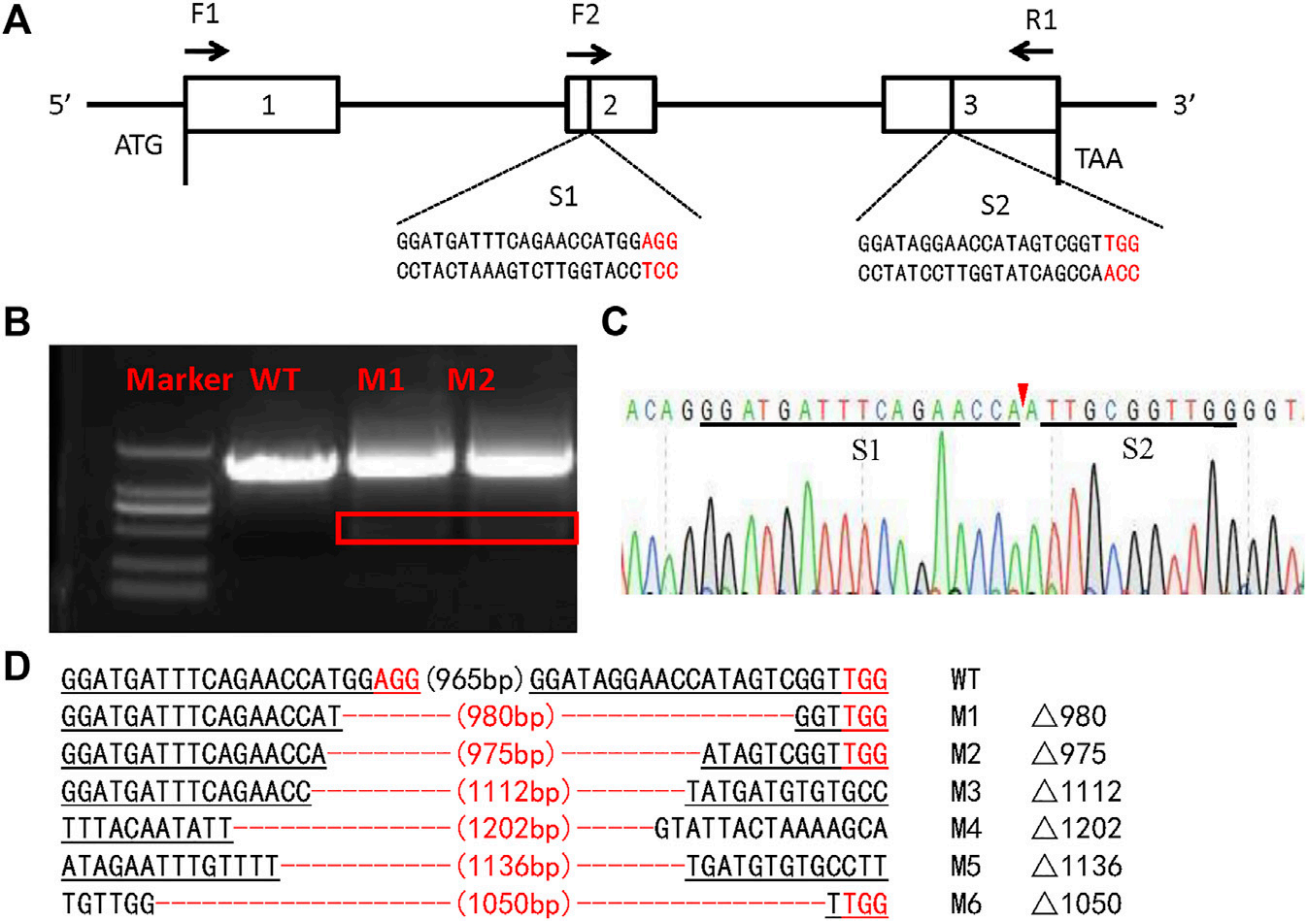
The schematic of *SlSer2* target sites and CRISPR/Cas9 mediated mutations. **(A)** Genomic structure of the *Ser2* gene in *Spodoptera litura* (*SlSer2*). The sgRNA targeting sequences (S1 and S2) are in black text and the protospacer adjacent motif (PAM) sequences are in red. The approximate locations of amplification primers (F2 and R1). **(B)** Agarose gel electrophoresis of PCR products used for initial detection of the mutations in the *SlSer2* gene. The red box represents the deleted sequence fragment of *SlSer2* mutants. **(C)** Sequencing chromatogram of *SlSer2* mutants. The black line represents the targeted region. The red wedge indicates position of cleavage by CRISPR/Cas9 genome editing system. **(D)** Mutations detected by sequencing. The PAM sequence is in red. The black line represents the target sites.

The *SlSer2* gene was cloned and sequenced by the Sanger sequencing method (Supplementary Figure S1). The 594-bp *SlSer2* gene consists of three exons and encodes a putative 197-amino acid protein (Figure 2A). Sequence analysis and multiple alignment showed SlSER2 protein has the trypsin-like serine protease domain, which is high conserved with other lepidopteran insects (Supplementary Figure S2). From qRT-PCR results, *SlSer2* gene was hardly expressed in larval stages, but its expression increased from pupal to adult stages (Figure 1A). Remarkably, *SlSer2* was most highly expressed in the male adult stage, which was similar to other lepidopteran insects, including *B. mori* (Xu et al., 2020), *P. xylostella* (Xu et al., 2020), and *H. cunea* (Li X. et al., 2022). Moreover, the *Ser2* gene was most highly expressed in the trachea, hindgut and testis (Figure 1B). The spatial and temporally specific expression of *SlSer2* provided a molecular basis for further functional analysis.

### CRISPR/Cas9 mediated the *Ser2* gene mutation in *Spodoptera litura*

To investigate the function of the *SlSer2* gene, we employed the CRISPR/Cas9 system to knockout this gene. Following the single guide RNA (sgRNA) design rule (Wang et al., 2013), we transcribed two sgRNAs *in vitro* targeting the second and third exons in the *Ser2* genome locus (Figure 2A). Using the embryo microinjection system, we injected 150 ng/μl for each sgRNA and 300 ng/μl Cas9 mRNA into eggs, laid less than 1 h before (Table 1). In order to detect the efficient of *SlSer2* sgRNAs timely, when these eggs hatched, the genomic DNA of the larvae was extracted and used Sanger sequencing to detect any mutated sequences. Sequencing chromatograms of the PCR product from injected eggs showed that sgRNAs of the *SlSer2* gene were effective (Figures 2B,C). Sequencing and mutagenesis analysis revealed that there were diverse deletion mutations of *SlSer2* genome sequences (Figure 2D).

**TABLE 1:** Mutagenesis of the *SlSer2* gene induced by injecting *Cas9* mRNA and sgRNA.

sgRNA for injection	sgRNA concentration (ng/μl)	Numbers of injected embryos	Larvae	Pupae	Adults
(T1+T2)*Ser2* sgRNAs	300	373	127 (34%)	89 (70%)	58 (65%)
EGFP sgRNA	300	236	90 (38%)	68 (76%)	48 (71%)

### Knockout of *SlSer2* results in male adult sterility

**FIGURE 3:**
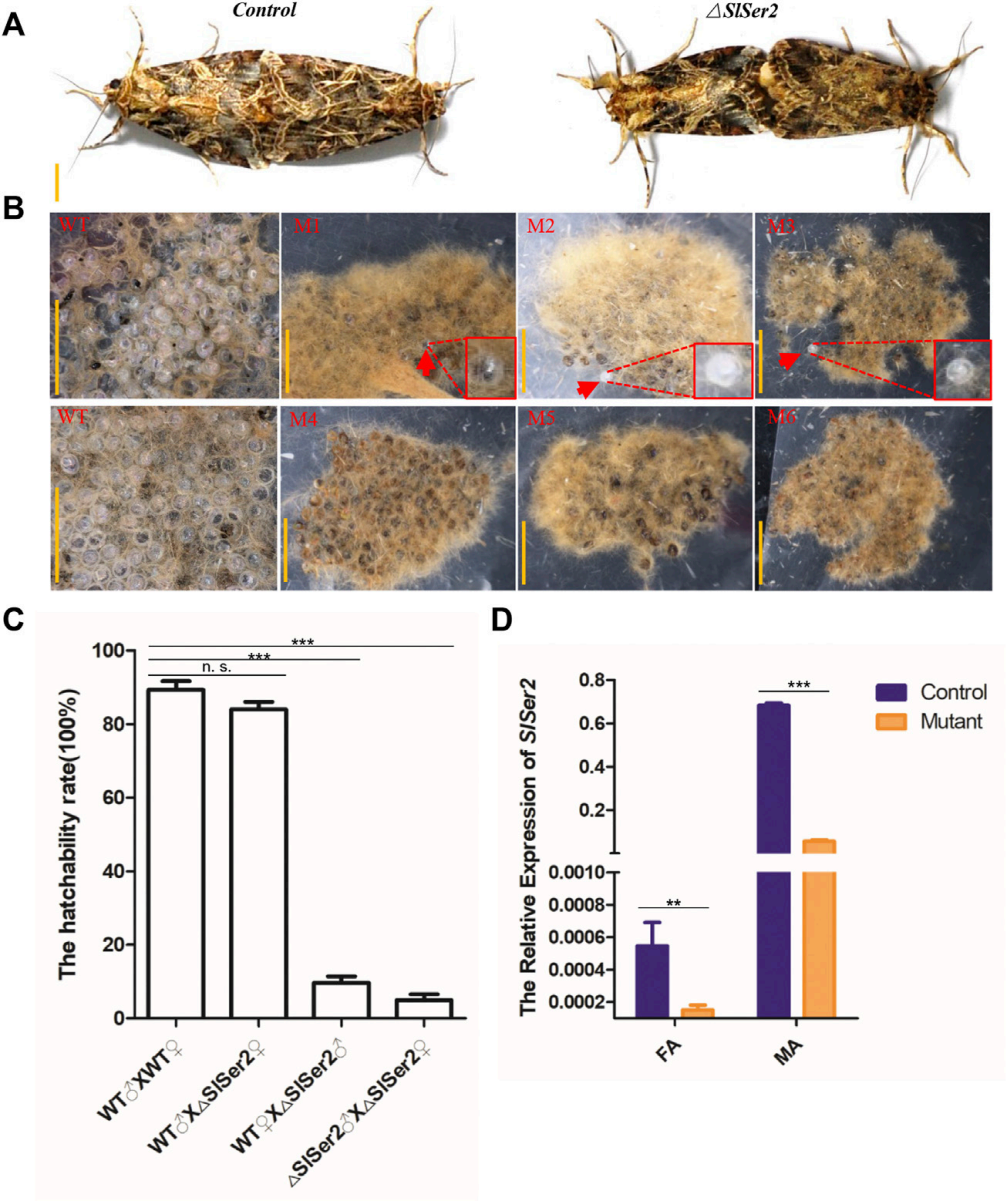
Loss of *SlSer2* function results in male sterility. **(A)** The wild type and *Ser2* mutants can mate with each other normally. Scale bar: 0.5 cm. **(B)** CRISPR/Cas9-mediated disruption of *Ser2* induced male sterility. WT, wild type egg masses. M1-M6, *Ser2* mutant egg masses. Scale bar: 2 mm. **(C)** The hatchability rate between wild type and mutants. **(D)** The relative mRNA expression of *SlSer2* in wild type and *Ser2* mutants. mRNA expression was normalized to *SlActin*. FA, female adult; MA, male adult. The data shown are means ± S.E.M. Asterisks indicate significant differences with a two-tailed *t*-test: *p < 0.05; **p < 0.01; ***p < 0.001; n. s. *p* > 0.05.

Considering the conserved function of *Ser2* gene linked to the male reproduction success in other lepidopteran insects (Xu et al., 2020; Li L. et al., 2022), we conducted the mating behavior analysis and hatchability assay. To identify the adult sterility of male mutants, we separated the male and female pupae of wild type and mutants respectively before the adult stage, which can prevent mating with each other and hold the virgin stage. When the hatched larvae grew into adults, a transparent plastic bag kept one pair group to mate with each other and lay eggs inside the plastic bag. The different mating groups included wild-type male mated with wild-type female, wild-type male mated with mutant female, wild-type female mated with mutant male and mutant male mated with mutant female. In both cases, the mating behavior was non-distinctive, and wild type and mutants of either sex could mate with each other successfully and rapidly (Figure 3A). However, the next generation of eggs produced by *Ser2* mutants were nearly all unhatched (Figure 3B). In the G0 generation, the wild type males could mate with wild type females and *△Ser2* females normally, and whether wild females or *△Ser2* females can lay normally hatched egg masses (Figure 3C). But when the *△Ser2* males mated with wild type females and *△Ser2* females, the hatching rate of egg masses produced by both types of females was lower (Figure 3C).

To confirm adult genotypes, we used qRT-PCR to detect relative expression of the *Ser2* gene in presumptive male and female mutants. These results showed that *Ser2* expression was significantly down-regulated in the presumptive mutants, compared with wild type males and females (Figure 3D). Furthermore, using the extracted genomic DNA from legs of mutants, PCR program-based sequencing results of the male and female individuals with the male-specific sterile genotypes, showed different deletion types in the *Ser2* loci (Supplementary Figure S3). These data demonstrated that disruption of the *SlSer2* gene can cause male-specific sterility in *S. litura*.

### Male sterility is stably heritable in the next generation

**FIGURE 4:**
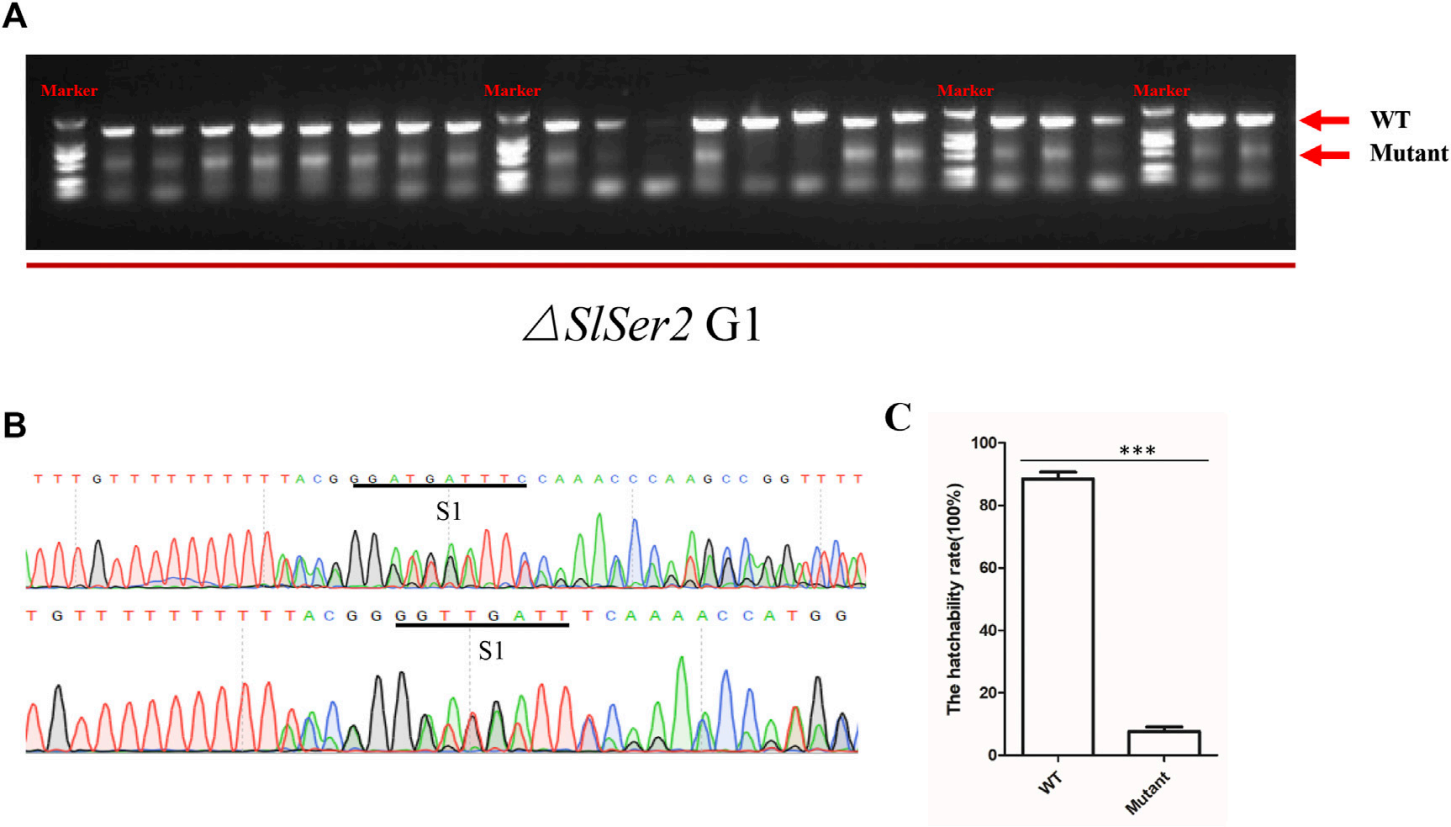
Male sterility is heritable stably in the next generation. **(A)** The PCR detection of mutations in G1 adult males. Arrows indicate band sizes. The upper major band is WT. Different size bands appear in each sample due to induction of different deletions and chimerism. **(B)** Representative sequencing chromatograms of PCR products of chimeric mutants. The bottom chromatogram shows peaks with multiple nucleotides at some positions (S1), indicating the occurrence of mutant and wild type sequences at the same loci. **(C)** The offspring hatchability rate of G1 male adult mutants. The data shown are means ± S.E.M. Asterisks indicate significant differences with a two-tailed *t*-test: ***p < 0.001.

In order to investigate the heritability of the phenotype of male sterility, *SlSer2* female mutants confirmed by PCR mated with wild type males. We found the progeny of the *SlSer2* female mutants could normally hatch and grow. Subsequently, when the next generation individuals developed to the adult stage, we extracted the DNA from each male insect and confirmed the mutant genotype through directed PCR (Figure 4A). Representative sequencing chromatograms of PCR products indicated that the mutants were chimeric. For example, the bottom chromatograms shown in Figure 4B have multiple peaks that indicate the occurrence of more than one nucleotide at a single locus. Ten male mutants mated with the wild type female adults to examine their reproductive status. The statistical result of hatchability showed that only about 10% eggs of female adults could hatch normally, which mated with male mutants and laid eggs (Figure 4C). Thus, these results showed that the male sterility induced by disrupting the *SlSer2* gene was stably heritable in the next generation.

### Mutation of the *SlSer2* gene affected the relative expression of some other genes

**FIGURE 5:**
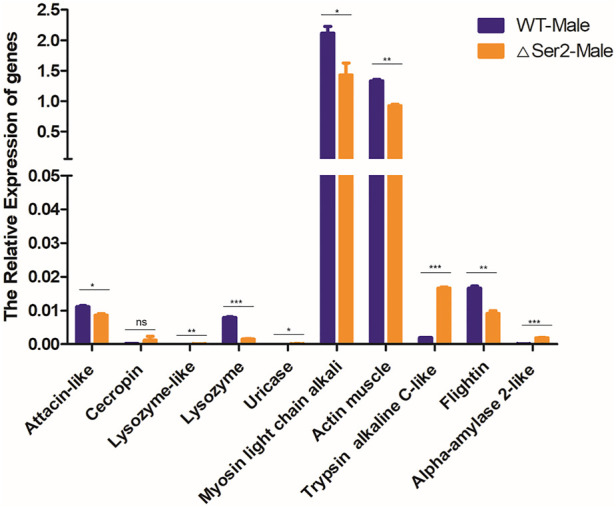
Mutation of the *SlSer2* gene affects relative expression of genes involved in adult stage. Levels of indicated mRNAs in *SlSer2* mutants relative to wild-type levels. Three individual biological replicates of real-time PCR were performed. The asterisks (** or ***) indicate the significant differences (*p* < 0.01 or *p* < 0.001, respectively) compared with the wild type adult with a two-tailed *t*-test.

To investigate the underlying reason for the *SlSer2* male sterility phenotypes, we used qRT-PCR to analyze the expression of a series of SFP genes involved in protein modification, motor proteins, and structural proteins, which shown to be critical for sperm function (Mcgraw et al., 2004). We selected some important function and potential regulation genes, including defense and immunity genes: *attacin-like* (XM_022981696.1), *cecropin* (XM_022971764.1); enzyme genes: *lysozyme-like* (XM_022959065.1), *lysozyme* (XM_022981495.1), *uricase* (XM_022965214.1); motor protein genes: *actin muscle* (XM_022981497.1), *myosin light chain alkali* (XM_022970316.1); protease genes: *trypsin alkaline C-like* (XM_022965904.1), *flightin* (XM_022976987.1) and calcium binding gene: *alpha-amylase 2-like* (XM_022958360.1) (Mcgraw et al., 2004). Compared with the wild type, the *Myosin light chain alkali* (*Mlc-A*) gene and *Actin muscle* (*Act-M*) gene, which are involved in the motor protein family, were down-regulated (Figure 5). The structural protein family gene *Flightin* was also significantly down-regulated compared with wild-type. In contrast, genes in the protease family, including the *Lysozyme-like* gene, *Uricase* gene and *Trypsin alkaline C-like* gene, were up-regulated in *Ser2* mutants. According to these qRT-PCR results, the *SlSer2* may regulate the sperm movement and activity to affect the male fertility in *S. litura*.

## Discussion

In this study, we investigated the function of the *Ser2* gene in the non-model insect *S. litura*. The *Ser2* gene belongs to the SFPs family. The SFPs family is vital to male fertility (Avila et al., 2011; Rodriguezmartinez et al., 2011; Laflamme et al., 2013) and plays a significant role in sperm activation and storage (Neubaum et al., 1999; Sirot et al., 2011; Nagaoka et al., 2012; Zhao et al., 2012); ovulation (Marshall et al., 2009; Xu et al., 2011); female immune function (Guerin et al., 2011), and post-mating behaviors in female insects (Sirot et al., 2009; Laflamme et al., 2012).

The *SlSer2* gene is 594-nucleotides long and consists of three exons (Figure 2). It encodes a putative 197-amino acid protein (Supplementary Figure S1). Multiple alignment showed that SlSER2 protein has the conservative Trypsin-like serine protease domain with 85% homology with other SER2 protein sequences (Supplementary Figure S2). We found that *SlSer2* was specifically expressed in the testis and adult male stage (Figure 1). As an important and high-efficiency genome editing technique, we successfully used the CRISPR/Cas9 system to knock out the *Ser2* gene in *S. litura* (Figure 2). Loss of function of the *SlSer2* gene induced the male sterility (Figure 3). In the progeny of *SlSer2* female mutants, we identified that male sterility was inherited stably (Figure 4). Using qRT-PCR, we detected changes in expression of some genes including members of a protease family, motor protein family and structural protein family (Figure 5). These results demonstrate the *SlSer2* is one of the most important SFPs involved in energy metabolism and proteolysis in *S. litura*. CRISPR/Cas9 disruption *Ser2* induced the male sterility without affecting female fertility. These phenotypes show *Ser2* is a potential target gene for pest control in *S. litura* and other lepidopteran insects.

The seminal fluid comprises the non-sperm component of the male ejaculate, which contains hundreds of proteins and non-protein components, and affects male fertility (Poiani et al., 2006; Avila et al., 2011; Rodriguezmartinez et al., 2011; Laflamme et al., 2013). Recent studies have shown that the SFPs are ubiquitous and have been identified in many species such as *Aedes aegypti* (Sirot et al., 2011), *Lutzomyia longipalpis* (Azevedo et al., 2012), *Ceratitis capitata* (Davies et al., 2006), *Apis mellifera* (Baer et al., 2009), *Heliconius erato* and *Heliconius melpomene* (Walters et al., 2008; Walters et al., 2010), *B. mori* (Nagaoka et al., 2012; Xu et al., 2020), *Tribolium castaneum* (South et al., 2011), *Gryllus firmus* and *Gryllus pennsylvanicus* (Andres et al., 2006) and *Amblyomma hebraeum* (Weiss et al., 2002). These reports show that SFPs are important components of insect male fertility. In SFPs, the serine proteases were the most common class of the protease (Laflamme et al., 2013), with a conserved catalytic triad consisting of a His, Ser, and Asp that coordinate a water molecule (Polgar et al., 1989). The prevalence of serine proteases in the seminal fluid was expected (Page et al., 2008). Moreover, in *D. melanogaster*, SFPs contribute to many biological processes including immune defense, protein modification, and metabolism (Swanson et al., 2002; Gillott et al., 2003; Mcgraw et al., 2004)*.* The qRT-PCR results of *SlSer2* mutations showed the motor protein family gene and structure protein family gene were down-regulated, and the protein modification family gene was up-regulated, suggesting that *SlSer2* involved in sperm movement and activity (Figure 5). In *B. mori* and *P. xylostella*, Xu et al (2020) used a transgenic CRISPR/Cas9 system to disrupt the *Ser2* gene and induce male sterility. In *H. cunea*, Li X. et al., 2022 used transgenic RNAi and CRISPR/Cas9 technologies to loss of function of *HcSer2* gene and demonstrated that *Ser2* is an essential and potential target gene for SIT. These results demonstrated that Serine proteases especially the *Ser2* gene, including *SlSer2*, have some conservative domains (Supplementary Figures S1, S2), expression manner (Figure 1) and played a vital function for male reproduction and sterility (Figures 3, 4).

The common cutworm, *S. litura* (Lepidoptera: Noctuidae) is one of the most destructive phytophagous pests (Tian et al., 2021; Tang et al., 2022). Pest management of lepidopteran insects is becoming increasingly difficult (Richardson et al., 2020; Li L. et al., 2022). SIT is an important approach for pest populations control. But choosing effective target genes is key to pest control by SIT (Harris et al., 2012). Our results suggest that the *Ser2* gene regulates the male reproductive capacity and has the potential as a better target gene for pest control, especially in *S. litura*. In the silkworm, *B. mori*, the seminal fluid protein genes have been identified as the useful target genes for SIT (Xu et al., 2020; Xu et al., 2022). Sustainability is an important factor in pest control. Disruption of the *Ser2* gene induced male sterility in the next generation and did not affect female reproduction. Thus, this study identified a useful sterile gene for pest control that may lead to control strategies in lepidopteran insect pests such as *S. litura*.

## Data Availability

The original contributions presented in the study are included in the article/Supplementary Materials, further inquiries can be directed to the corresponding authors.
