# Supplementary material for: CRISPR/Cas9-mediated Serine protease 2 disruption induces male sterility in Spodoptera litura
Source: Front Physiol. 2022 Aug 3;13:931824. doi: 10.3389/fphys.2022.931824 (PMC9382020; doi:10.3389/fphys.2022.931824)
Supplement: Supplementary file 1 [file Datasheet1.docx]

**Figure S1.** Nucleotide and deduced amino acid sequences of *S. litura Ser2*. The black box and red box represent the ATG and stop codons, respectively. The blue line represents the Trypsin-like serine protease domain (Tryp_SPc).


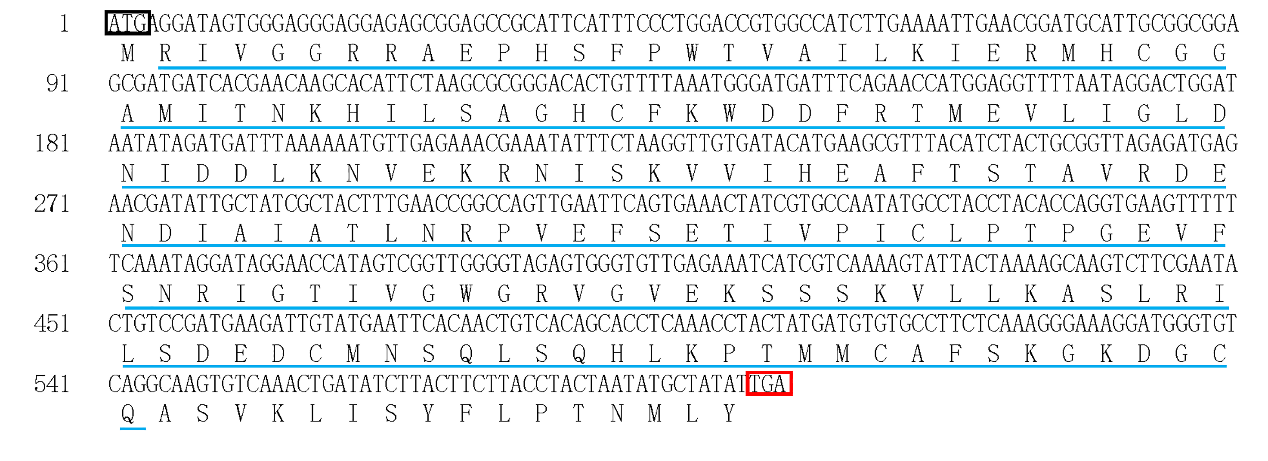


**Figure S2.** Multiple alignment of SER2 Protein. The GenBank accession numbers of the protein sequence are as follows: *Bombyx mori* (NP_001153675.1), *Agrius* *convolvuli* (BAK52270.1), *Samia* *ricini* (BAL04890.1), *Pieris* *rapae* (XP_022113521.1), *Helicoverpa* *armigera* (XP_021195380.1), *Papilio* *machaon* (XP_014359308.1), *Plutella* *xylostella* (XP_011553524.1), *Hyphantria* *cunea* (Li L. et al., 2022) and the putative SER2 protein of *S. litura* (UTN00747.1)*.*


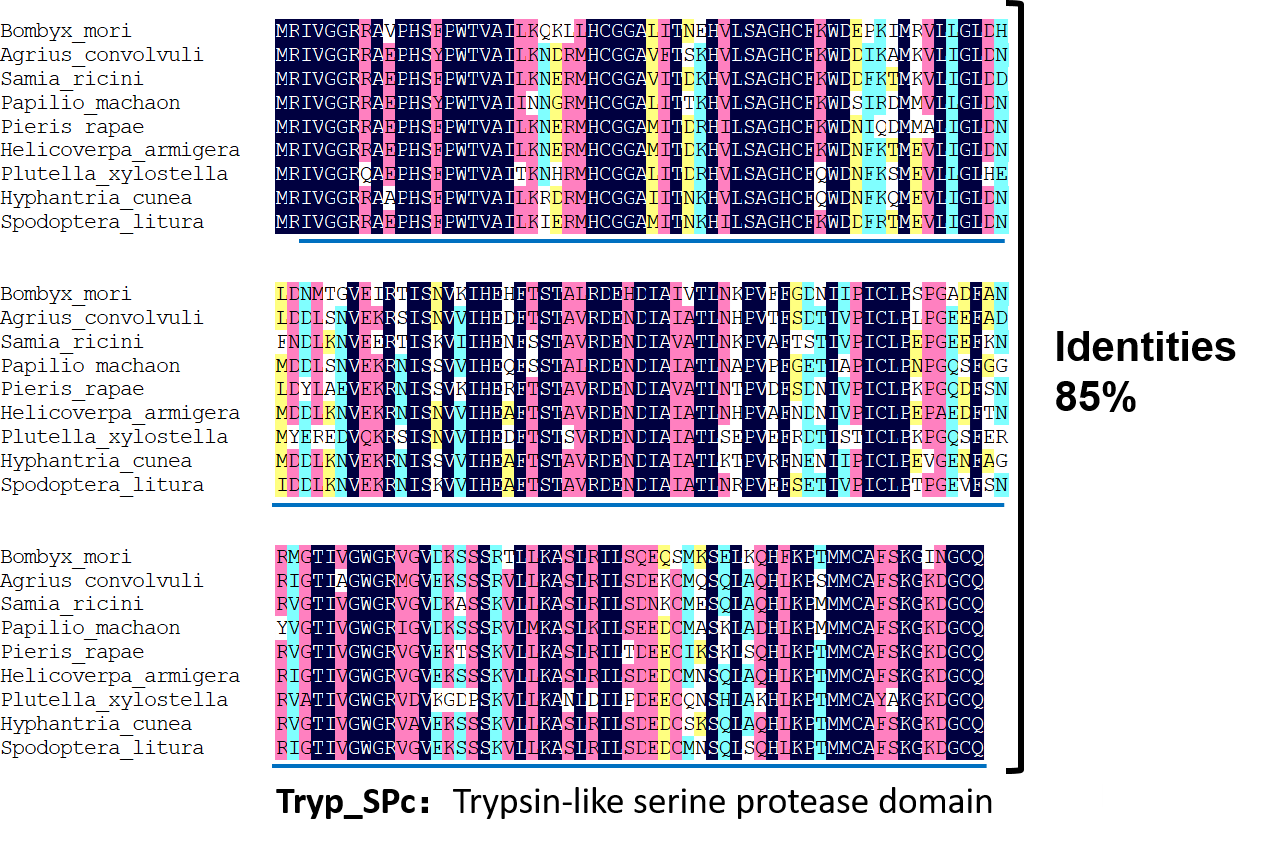


**Figure S3.** Genomic sequence changes in G0 mutants

1. Agarose gel electrophoresis of PCR products using *Ser2* gene specific primers with DNA samples of G0 adults. Different size bands appear in each sample due to induction of different deletions and chimerism.
2. Sanger sequencing chromatograms of independent mutants revealing large genome sequence deletions marked by underlines. The arrows mark the cutting position of CRISPR/Cas9 system in the targeted sequence.


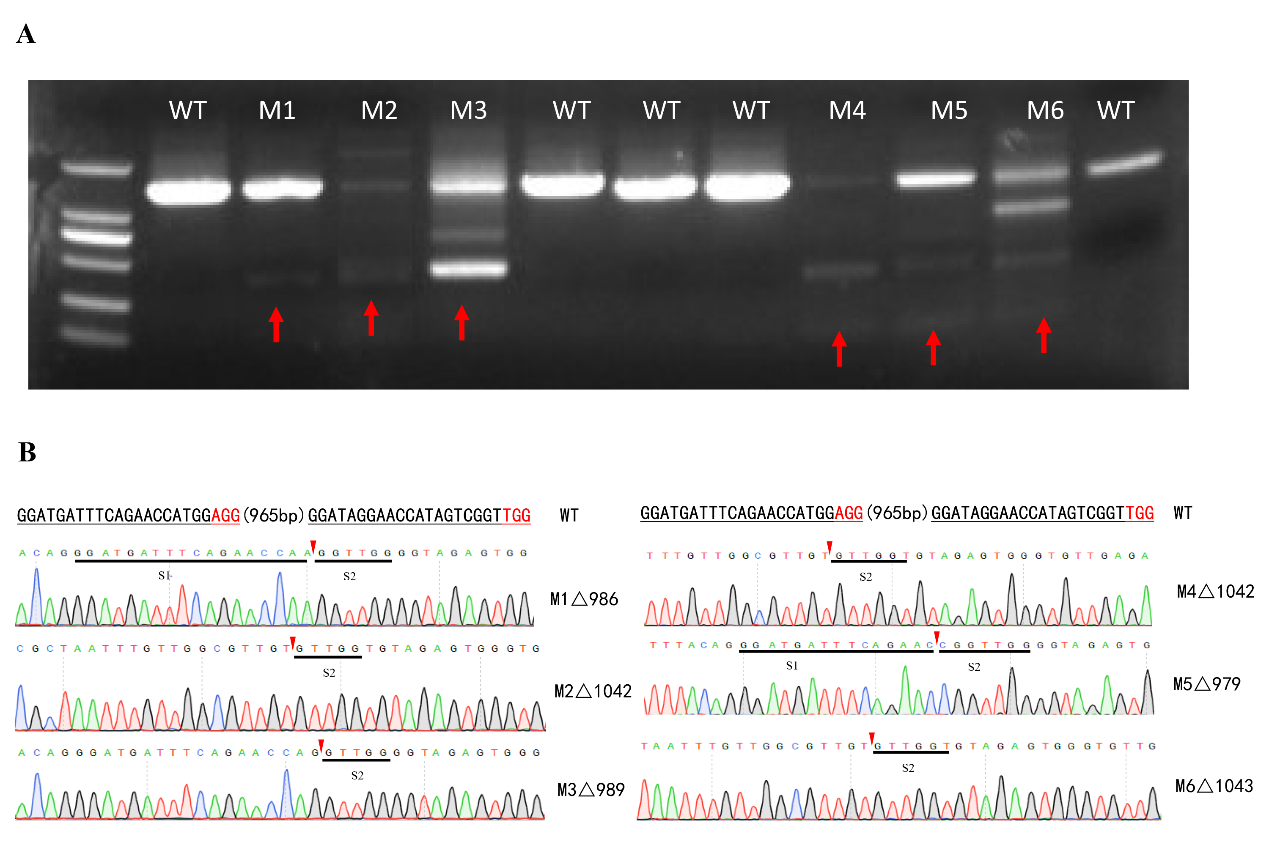


**Table S1**. The component of artificial diet in *Ostrinia furnacalis*

| Components | Weight (g) or volume (ml) |
| --- | --- |
| Wheat germ powder | 150 |
| Yeast extract powder | 40 |
| Agar strip | 14 |
| Sucrose | 5 |
| Vitamin C | 4 |
| Sorbic Acid | 4 |
| Methyl p-hydroxybenzoate | 4 |
| Linoleic acid | 0.5 |
| purified water | 900 |

Preparation method: Boil 900 ml of water mixed with agar, add other ingredients, mix well, and store in the refrigerator after cooling.

**Table S2**. Primers used in this study.

| Primer name | Primer sequence(5'-3') | Primer purpose |
| --- | --- | --- |
| Ser2-sgF1 | TAATACGACTCACTATAGGATGATTTCAGAACCATGG  GTTTTAGAGCTAGAAATAGCAAGTTAAAATAAG | Preparation of sgRNA templates |
| Ser2-sgF2 | TAATACGACTCACTATAGGATAGGAACCATAGTCGGT  GTTTTAGAGCTAGAAATAGCAAGTTAAAATAAG | Preparation of sgRNA templates |
| EGFP-sgF1 | TAATACGACTCACTATAGGGCGAGGAGCTGTTCACCG  GTTTTAGAGCTAGAAATAGCAAGTTAAAATAAG | Preparation of sgRNA templates |
| EGFP-sgF2 | TAATACGACTCACTATAGGCCACAAGTTCAGCGTGTC  GTTTTAGAGCTAGAAATAGCAAGTTAAAATAAG | Preparation of sgRNA templates |
| sgRNA-R | AAAAGCACCGACTCGGTGCCACTTTTTCAAGTTGATAA  CGGACTAGCCTTATTTTAACTTGCTATTTCT | Preparation of sgRNA templates |
| F1 | ATGAGGATAGTGGGAGGGAGGAGAGCGG | Identification of somatic mutations |
| F2 | TTTCGTGAACTGACACTCTATTTCGA | Identification of somatic mutations |
| R1 | CTTCTTACCTACTAATATGCTATATTGA | Identification of somatic mutations |
| Ser2-qF | ATCGTGCCAATATGCCTACC | qPCR |
| Ser2-qR | TTCCCTTTGAGAAGGCACAC | qPCR |
| Actin-qF | TCCTGGACTCCGGTGATGGTGT | qPCR |
| Actin-qR | CAGCGGTGGTGGTGAAAGAGTAAC | qPCR |
| Attacin-like-F | GTGGGAAACAGTCCCAAGTC | qPCR |
| Attacin-like-R | AGCTCCTCCGATAGCACTGA | qPCR |
| Cecropin-F | TAATGTCCACCGTGTCAGGA | qPCR |
| Cecropin-R | AACTGCTGGTCCTGCCTTTA | qPCR |
| Lysozyme-like-F | TAGTGAAAGTGCTCGCCACA | qPCR |
| Lysozyme-like-R | CTTGGCGCCTGAAGATTTTA | qPCR |
| Lysozyme-F | ACATACTTCCCAGGGTCACG | qPCR |
| Lysozyme-R | GCACCAATCCTTGTTGTTGA | qPCR |
| Uricase-F | GGAAGTACCACCCTCAAGCA | qPCR |
| Uricase-R | TATGCAGTTTCGGAGTGCAG | qPCR |
| Myosin light chain alkali-F | CAGCAAGAACGACGTTGAAA | qPCR |
| Myosin light chain alkali-F | CTTGGCTGTAGATGGGAAGG | qPCR |
| Actin muscle-F | CGTTCGTGACATCAAGGAGA | qPCR |
| Actin muscle-R | GGATACCGCAGGATTCCATA | qPCR |
| Trypsin alkaline C-like-F | TTGACCAGTACCCCACCATT | qPCR |
| Trypsin alkaline C-like-R | GGTTAGCGTTGACGTTGTGA | qPCR |
| Flightin-F | GCACCGAACCCAAGAAACTA | qPCR |
| Flightin-R | TGGAAAGGTAGGTCCTGAGC | qPCR |
| Alpha-amylase 2-like-F | ATGAGCTCCTTCGACTTCCA | qPCR |
| Alpha-amylase 2-like-R | CTACCGTTGTCCCACCAGTT | qPCR |
